# 5-(4-Pyrid­yl)-1,3,4-thia­diazol-2-amine

**DOI:** 10.1107/S1600536809014470

**Published:** 2009-04-25

**Authors:** Yao Wang, Rong Wan, Feng Han, Peng Wang, Bin Wang

**Affiliations:** a Department of Applied Chemistry, College of Science, Nanjing University of Technology, No. 5 Xinmofan Road, Nanjing, Nanjing 210009, People’s Republic of China

## Abstract

The title compound, C_7_H_6_N_4_S, was synthesized by reacting pyridine-4-carboxylic acid and thio­semicarbazide. The asymmetric unit contains two independent mol­ecules, which present different conformations, the dihedral angles between the thia­diazole and pyridine rings being 18.2 (2) and 30.3 (2)°. In the crystal, inter­molecular N—H⋯N hydrogen bonds involving the amine groups as donors link mol­ecules into a two-dimensional framework.

## Related literature

For the biological activity of 1,3,4-thia­diazo­les, see: Nakagawa *et al.* (1996 ▶); Wang *et al.* (1999 ▶). For the structure of 2-amino-5-phenyl-1,3,4-thia­diazole, see: Öztürk *et al.* (2004 ▶).
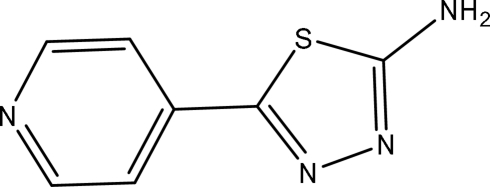


## Experimental

### 

#### Crystal data

C_7_H_6_N_4_S
*M* *_r_* = 178.22
Monoclinic, 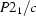

*a* = 14.794 (3) Å
*b* = 10.686 (2) Å
*c* = 10.477 (2) Å
β = 106.52 (3)°
*V* = 1587.9 (5) Å^3^
*Z* = 8
Mo *K*α radiation
μ = 0.35 mm^−1^
*T* = 293 K
0.20 × 0.10 × 0.10 mm

#### Data collection

Enraf–Nonius CAD-4 diffractometer
Absorption correction: ψ scan (North *et al.*, 1968 ▶) *T*_min_ = 0.933, *T*_max_ = 0.966
3203 measured reflections
3023 independent reflections
1516 reflections with *I* > 2σ(*I*)
*R*_int_ = 0.042
3 standard reflections every 200 reflections intensity decay: 1%

#### Refinement

*R*[*F* ^2^ > 2σ(*F* ^2^)] = 0.067
*wR*(*F* ^2^) = 0.142
*S* = 1.00
3023 reflections
217 parameters
43 restraints
H-atom parameters constrained
Δρ_max_ = 0.24 e Å^−3^
Δρ_min_ = −0.31 e Å^−3^

### 

Data collection: *CAD-4 EXPRESS* (Enraf–Nonius, 1989 ▶); cell refinement: *CAD-4 EXPRESS*; data reduction: *XCAD4* (Harms & Wocadlo,1995 ▶); program(s) used to solve structure: *SHELXS97* (Sheldrick, 2008 ▶); program(s) used to refine structure: *SHELXL97* (Sheldrick, 2008 ▶); molecular graphics: *SHELXTL* (Sheldrick, 2008 ▶); software used to prepare material for publication: *SHELXL97*.

## Supplementary Material

Crystal structure: contains datablocks global, I. DOI: 10.1107/S1600536809014470/bh2224sup1.cif

Structure factors: contains datablocks I. DOI: 10.1107/S1600536809014470/bh2224Isup2.hkl

Additional supplementary materials:  crystallographic information; 3D view; checkCIF report

## Figures and Tables

**Table 1:** Hydrogen-bond geometry (Å, °)

*D*—H⋯*A*	*D*—H	H⋯*A*	*D*⋯*A*	*D*—H⋯*A*
N4*A*—H4*A*⋯N1*B*^i^	0.86	2.08	2.940 (5)	177
N4*A*—H4*B*⋯N2*A*^ii^	0.86	2.21	3.053 (5)	166
N4*B*—H8*A*⋯N1*A*^iii^	0.86	2.10	2.945 (5)	168
N4*B*—H8*B*⋯N2*B*^iv^	0.86	2.13	2.988 (5)	178

Symmetry codes: (i) 
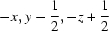
; (ii) 
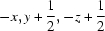
; (iii) 
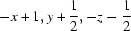
; (iv) 
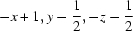
.

**Fig. 1.**
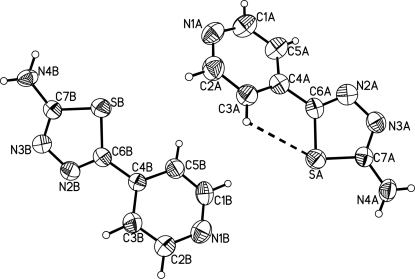
A view of the molecular structure of the title compound. Displacement ellipsoids are drawn at the 50% probability level. The dashed line indicates an intramolecular C—H···S hydrogen bond.

**Fig. 2.**
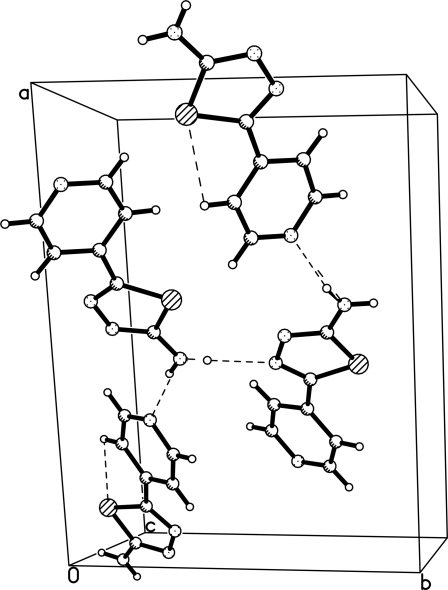
Partial packing view showing the hydrogen bonds network. Dashed lines indicate intermolecular N—H···N hydrogen bonds.

## supplementary crystallographic information

### Comment

1,3,4-Thiadiazole derivatives represent an interesting class of compounds possessing a broad spectrum of biological activity (Nakagawa *et al.*, 1996). These compounds are known to exhibit diverse biological effects, such as insecticidal and fungicidal activities (Wang *et al.*, 1999).

The asymmetric unit of the title compound contains two independent molecules (A and B, see Fig. 1), with bond lengths and angles in expected ranges. (Öztürk *et al.*, 2004). Dihedral angles between thiadiazole and pyridine rings are different in each molecule: 18.2 (2)° for molecule A and 30.3 (2)° in molecule B. In the crystal, molecules are linked through N—H···N hydrogen bonds, forming a two-dimensional supramolecular structure.

### Experimental

4-Pyridinecarboxylic acid (2 mmol) and thiosemicarbazide (5 mmol) were mixed in a 25 ml flask, and kept in the oil bath at 363 K for 6 h. After cooling, the crude product precipitated and was filtered. Pure compound was obtained by crystallization from ethanol. Crystals suitable for X-ray diffraction were obtained by slow evaporation of an acetone solution.

### Refinement

All H atoms were placed geometrically with C—H and N—H bond lengths fixed to 0.93 and 0.86 Å, respectively, and included in the refinement in the riding motion approximation, with *U*_iso_(H) = 1.2 *U*_eq_(carrier atom). In the B molecule, displacement parameters for atoms C3B/C4B/C5B/C6B/N4B/C7B were restrained to approximate an isotropic behaviour, and a rigid bond restraint was applied to fragments C3B/C4B/C5B/C6B and N4B C7B.

### Crystal data

**Table:**

C_7_H_6_N_4_S	*F*(000) = 736
*M**_r_* = 178.22	*D*_x_ = 1.491 Mg m^−^^3^
Monoclinic, *P*2_1_/*c*	Melting point: 543 K
Hall symbol: -P 2ybc	Mo *K*α radiation, λ = 0.71073 Å
*a* = 14.794 (3) Å	Cell parameters from 25 reflections
*b* = 10.686 (2) Å	θ = 9–12°
*c* = 10.477 (2) Å	µ = 0.35 mm^−^^1^
β = 106.52 (3)°	*T* = 293 K
*V* = 1587.9 (5) Å^3^	Block, colorless
*Z* = 8	0.20 × 0.10 × 0.10 mm

### Data collection

**Table:**

Enraf–Nonius CAD-4 diffractometer	1516 reflections with *I* > 2σ(*I*)
Radiation source: fine-focus sealed tube	*R*_int_ = 0.042
graphite	θ_max_ = 26.0°, θ_min_ = 1.4°
ω/2θ scans	*h* = −18→17
Absorption correction: ψ scan (North *et al.*, 1968)	*k* = −13→0
*T*_min_ = 0.933, *T*_max_ = 0.966	*l* = 0→12
3203 measured reflections	3 standard reflections every 200 reflections
3023 independent reflections	intensity decay: 1%

### Refinement

**Table:**

Refinement on *F*^2^	Primary atom site location: structure-invariant direct methods
Least-squares matrix: full	Secondary atom site location: difference Fourier map
*R*[*F*^2^ > 2σ(*F*^2^)] = 0.067	Hydrogen site location: inferred from neighbouring sites
*wR*(*F*^2^) = 0.142	H-atom parameters constrained
*S* = 1.00	*w* = 1/[σ^2^(*F*_o_^2^) + (0.0435*P*)^2^] where *P* = (*F*_o_^2^ + 2*F*_c_^2^)/3
3023 reflections	(Δ/σ)_max_ < 0.001
217 parameters	Δρ_max_ = 0.24 e Å^−^^3^
43 restraints	Δρ_min_ = −0.31 e Å^−^^3^
0 constraints	

### Fractional atomic coordinates and isotropic or equivalent isotropic displacement parameters (Å^2^)

**Table:**

	*x*	*y*	*z*	*U*_iso_*/*U*_eq_	
SA	0.08251 (8)	0.54450 (10)	0.19829 (12)	0.0483 (4)	
N1A	0.3034 (3)	0.3300 (4)	−0.0749 (4)	0.0551 (11)	
N2A	0.0328 (3)	0.3203 (3)	0.1338 (4)	0.0513 (10)	
N3A	−0.0258 (2)	0.3605 (3)	0.2081 (4)	0.0509 (10)	
N4A	−0.0553 (2)	0.5382 (3)	0.3223 (3)	0.0548 (11)	
H4A	−0.0999	0.5013	0.3454	0.066*	
H4B	−0.0408	0.6143	0.3462	0.066*	
C1A	0.2262 (3)	0.2621 (5)	−0.0932 (4)	0.0604 (14)	
H1B	0.2192	0.1944	−0.1510	0.073*	
C2A	0.3105 (3)	0.4259 (5)	0.0056 (5)	0.0601 (14)	
H2B	0.3635	0.4769	0.0204	0.072*	
C3A	0.2430 (3)	0.4551 (4)	0.0697 (4)	0.0485 (12)	
H3B	0.2515	0.5243	0.1256	0.058*	
C4A	0.1643 (3)	0.3827 (4)	0.0511 (4)	0.0393 (11)	
C5A	0.1561 (3)	0.2820 (4)	−0.0357 (4)	0.0549 (13)	
H5A	0.1038	0.2296	−0.0538	0.066*	
C6A	0.0928 (3)	0.4040 (4)	0.1217 (4)	0.0413 (11)	
C7A	−0.0070 (3)	0.4754 (4)	0.2470 (4)	0.0396 (11)	
SB	0.43176 (9)	0.68957 (10)	−0.17070 (12)	0.0505 (4)	
N1B	0.2053 (3)	0.9156 (4)	0.0901 (4)	0.0573 (11)	
N2B	0.4342 (3)	0.9258 (3)	−0.1864 (4)	0.0518 (11)	
N3B	0.4926 (2)	0.8856 (3)	−0.2569 (4)	0.0489 (10)	
N4B	0.5574 (2)	0.7052 (3)	−0.3157 (3)	0.0456 (10)	
H8A	0.5907	0.7475	−0.3556	0.055*	
H8B	0.5607	0.6249	−0.3136	0.055*	
C1B	0.1980 (3)	0.8094 (5)	0.0207 (5)	0.0562 (13)	
H8C	0.1499	0.7540	0.0226	0.067*	
C2B	0.2762 (3)	0.9914 (4)	0.0858 (4)	0.0577 (13)	
H9A	0.2837	1.0650	0.1352	0.069*	
C3B	0.3375 (3)	0.9687 (4)	0.0150 (4)	0.0507 (12)	
H10A	0.3841	1.0269	0.0141	0.061*	
C4B	0.3308 (3)	0.8588 (4)	−0.0563 (4)	0.0362 (10)	
C5B	0.2573 (3)	0.7782 (4)	−0.0528 (4)	0.0506 (12)	
H12A	0.2489	0.7035	−0.1003	0.061*	
C6B	0.3977 (3)	0.8349 (4)	−0.1355 (4)	0.0389 (10)	
C7B	0.4998 (3)	0.7650 (4)	−0.2563 (4)	0.0380 (10)	

### Atomic displacement parameters (Å^2^)

**Table:**

	*U*^11^	*U*^22^	*U*^33^	*U*^12^	*U*^13^	*U*^23^
SA	0.0477 (7)	0.0469 (7)	0.0499 (8)	−0.0047 (6)	0.0134 (6)	−0.0041 (6)
N1A	0.054 (3)	0.071 (3)	0.041 (2)	0.013 (2)	0.015 (2)	−0.002 (2)
N2A	0.045 (2)	0.057 (2)	0.047 (2)	−0.001 (2)	0.005 (2)	−0.003 (2)
N3A	0.046 (2)	0.049 (2)	0.056 (3)	−0.0055 (19)	0.012 (2)	0.003 (2)
N4A	0.058 (3)	0.065 (3)	0.050 (3)	−0.012 (2)	0.028 (2)	0.000 (2)
C1A	0.054 (3)	0.080 (4)	0.043 (3)	−0.004 (3)	0.006 (3)	−0.024 (3)
C2A	0.043 (3)	0.067 (4)	0.071 (4)	0.004 (3)	0.018 (3)	0.011 (3)
C3A	0.046 (3)	0.052 (3)	0.046 (3)	−0.003 (2)	0.011 (2)	−0.004 (2)
C4A	0.029 (2)	0.050 (3)	0.031 (3)	0.003 (2)	−0.006 (2)	0.006 (2)
C5A	0.041 (3)	0.076 (4)	0.041 (3)	−0.001 (3)	0.001 (2)	−0.014 (3)
C6A	0.036 (3)	0.048 (3)	0.037 (3)	−0.004 (2)	0.004 (2)	0.007 (2)
C7A	0.035 (2)	0.046 (3)	0.032 (2)	0.009 (2)	0.002 (2)	0.012 (2)
SB	0.0587 (8)	0.0404 (6)	0.0564 (8)	0.0000 (6)	0.0229 (7)	0.0013 (6)
N1B	0.048 (3)	0.069 (3)	0.055 (3)	0.010 (2)	0.015 (2)	0.007 (2)
N2B	0.056 (3)	0.038 (2)	0.065 (3)	0.0007 (19)	0.022 (2)	0.003 (2)
N3B	0.049 (2)	0.045 (2)	0.055 (3)	0.0003 (19)	0.019 (2)	−0.004 (2)
N4B	0.063 (2)	0.036 (2)	0.047 (2)	0.0107 (18)	0.031 (2)	0.0063 (18)
C1B	0.039 (3)	0.069 (3)	0.060 (3)	−0.009 (3)	0.013 (3)	0.007 (3)
C2B	0.063 (3)	0.058 (3)	0.057 (3)	−0.006 (3)	0.025 (3)	−0.004 (3)
C3B	0.046 (3)	0.055 (3)	0.053 (3)	−0.008 (2)	0.017 (2)	−0.007 (2)
C4B	0.031 (2)	0.039 (2)	0.034 (2)	0.0008 (19)	0.002 (2)	−0.001 (2)
C5B	0.047 (3)	0.046 (3)	0.059 (3)	−0.002 (2)	0.016 (2)	0.001 (2)
C6B	0.035 (2)	0.042 (2)	0.034 (2)	−0.001 (2)	0.001 (2)	0.001 (2)
C7B	0.033 (2)	0.037 (2)	0.036 (2)	0.002 (2)	−0.003 (2)	0.005 (2)

### Geometric parameters (Å, °)

**Table:**

SA—C7A	1.716 (4)	SB—C6B	1.705 (4)
SA—C6A	1.729 (4)	SB—C7B	1.726 (4)
N1A—C2A	1.312 (5)	N1B—C1B	1.335 (5)
N1A—C1A	1.320 (5)	N1B—C2B	1.336 (5)
N2A—C6A	1.292 (5)	N2B—C6B	1.298 (5)
N2A—N3A	1.387 (4)	N2B—N3B	1.356 (4)
N3A—C7A	1.298 (5)	N3B—C7B	1.293 (5)
N4A—C7A	1.380 (5)	N4B—C7B	1.349 (4)
N4A—H4A	0.8600	N4B—H8A	0.8600
N4A—H4B	0.8600	N4B—H8B	0.8600
C1A—C5A	1.356 (6)	C1B—C5B	1.363 (6)
C1A—H1B	0.9300	C1B—H8C	0.9300
C2A—C3A	1.389 (6)	C2B—C3B	1.347 (5)
C2A—H2B	0.9300	C2B—H9A	0.9300
C3A—C4A	1.365 (5)	C3B—C4B	1.380 (5)
C3A—H3B	0.9300	C3B—H10A	0.9300
C4A—C5A	1.392 (5)	C4B—C5B	1.396 (5)
C4A—C6A	1.470 (5)	C4B—C6B	1.483 (5)
C5A—H5A	0.9300	C5B—H12A	0.9300
			
C7A—SA—C6A	86.7 (2)	C6B—SB—C7B	86.5 (2)
C2A—N1A—C1A	115.6 (4)	C1B—N1B—C2B	116.1 (4)
C6A—N2A—N3A	113.4 (4)	C6B—N2B—N3B	113.0 (3)
C7A—N3A—N2A	110.9 (3)	C7B—N3B—N2B	112.2 (4)
C7A—N4A—H4A	120.0	C7B—N4B—H8A	120.0
C7A—N4A—H4B	120.0	C7B—N4B—H8B	120.0
H4A—N4A—H4B	120.0	H8A—N4B—H8B	120.0
N1A—C1A—C5A	126.0 (5)	N1B—C1B—C5B	123.4 (4)
N1A—C1A—H1B	117.0	N1B—C1B—H8C	118.3
C5A—C1A—H1B	117.0	C5B—C1B—H8C	118.3
N1A—C2A—C3A	123.2 (5)	N1B—C2B—C3B	124.5 (5)
N1A—C2A—H2B	118.4	N1B—C2B—H9A	117.7
C3A—C2A—H2B	118.4	C3B—C2B—H9A	117.7
C4A—C3A—C2A	120.4 (4)	C2B—C3B—C4B	119.7 (4)
C4A—C3A—H3B	119.8	C2B—C3B—H10A	120.2
C2A—C3A—H3B	119.8	C4B—C3B—H10A	120.2
C3A—C4A—C5A	116.3 (4)	C3B—C4B—C5B	116.6 (4)
C3A—C4A—C6A	123.2 (4)	C3B—C4B—C6B	119.5 (4)
C5A—C4A—C6A	120.5 (4)	C5B—C4B—C6B	123.9 (4)
C1A—C5A—C4A	118.5 (5)	C1B—C5B—C4B	119.7 (4)
C1A—C5A—H5A	120.8	C1B—C5B—H12A	120.2
C4A—C5A—H5A	120.8	C4B—C5B—H12A	120.2
N2A—C6A—C4A	123.7 (4)	N2B—C6B—C4B	121.5 (4)
N2A—C6A—SA	113.7 (3)	N2B—C6B—SB	114.2 (3)
C4A—C6A—SA	122.6 (3)	C4B—C6B—SB	124.3 (3)
N3A—C7A—N4A	122.7 (4)	N3B—C7B—N4B	122.1 (4)
N3A—C7A—SA	115.3 (3)	N3B—C7B—SB	114.1 (3)
N4A—C7A—SA	121.9 (3)	N4B—C7B—SB	123.8 (3)
			
C6A—N2A—N3A—C7A	0.9 (5)	C6B—N2B—N3B—C7B	−0.7 (6)
C2A—N1A—C1A—C5A	−1.0 (7)	C2B—N1B—C1B—C5B	−0.9 (7)
C1A—N1A—C2A—C3A	0.8 (7)	C1B—N1B—C2B—C3B	1.6 (7)
N1A—C2A—C3A—C4A	0.1 (7)	N1B—C2B—C3B—C4B	−2.1 (8)
C2A—C3A—C4A—C5A	−1.0 (6)	C2B—C3B—C4B—C5B	1.7 (6)
C2A—C3A—C4A—C6A	176.4 (4)	C2B—C3B—C4B—C6B	179.3 (4)
N1A—C1A—C5A—C4A	0.1 (8)	N1B—C1B—C5B—C4B	0.7 (7)
C3A—C4A—C5A—C1A	0.9 (6)	C3B—C4B—C5B—C1B	−1.1 (6)
C6A—C4A—C5A—C1A	−176.6 (4)	C6B—C4B—C5B—C1B	−178.6 (4)
N3A—N2A—C6A—C4A	178.1 (4)	N3B—N2B—C6B—C4B	−179.1 (4)
N3A—N2A—C6A—SA	−1.2 (5)	N3B—N2B—C6B—SB	−0.4 (5)
C3A—C4A—C6A—N2A	−160.7 (4)	C3B—C4B—C6B—N2B	−29.4 (6)
C5A—C4A—C6A—N2A	16.7 (6)	C5B—C4B—C6B—N2B	147.9 (4)
C3A—C4A—C6A—SA	18.5 (6)	C3B—C4B—C6B—SB	152.0 (3)
C5A—C4A—C6A—SA	−164.1 (3)	C5B—C4B—C6B—SB	−30.7 (6)
C7A—SA—C6A—N2A	0.9 (3)	C7B—SB—C6B—N2B	1.0 (3)
C7A—SA—C6A—C4A	−178.4 (4)	C7B—SB—C6B—C4B	179.7 (4)
N2A—N3A—C7A—N4A	179.9 (4)	N2B—N3B—C7B—N4B	−177.2 (4)
N2A—N3A—C7A—SA	−0.2 (5)	N2B—N3B—C7B—SB	1.5 (5)
C6A—SA—C7A—N3A	−0.4 (3)	C6B—SB—C7B—N3B	−1.4 (3)
C6A—SA—C7A—N4A	179.6 (4)	C6B—SB—C7B—N4B	177.3 (4)

### Hydrogen-bond geometry (Å, °)

**Table:**

*D*—H···*A*	*D*—H	H···*A*	*D*···*A*	*D*—H···*A*
C3A—H3B···SA	0.93	2.82	3.191 (5)	105
N4A—H4A···N1B^i^	0.86	2.08	2.940 (5)	177
N4A—H4B···N2A^ii^	0.86	2.21	3.053 (5)	166
N4B—H8A···N1A^iii^	0.86	2.10	2.945 (5)	168
N4B—H8B···N2B^iv^	0.86	2.13	2.988 (5)	178

Symmetry codes: (i) −*x*, *y*−1/2, −*z*+1/2; (ii) −*x*, *y*+1/2, −*z*+1/2; (iii) −*x*+1, *y*+1/2, −*z*−1/2; (iv) −*x*+1, *y*−1/2, −*z*−1/2.
